# Anticancer, anti-inflammatory and analgesic activities of aminoalcohol-based quinoxaline small molecules

**DOI:** 10.1590/acb395124

**Published:** 2024-08-05

**Authors:** Jannyely Moreira Neri, Paula Emília Apolônio Siqueira, Ana Luiza Cabral de Sá Leitão Oliveira, Renata Mendonça Araújo, Raimundo Fernandes de Araújo, Agnes Andrade Martins, Isabelle de Lima Marques, Rafaela Alcindo Silva, Aurigena Antunes de Araújo, Fabrício Gava Menezes

**Affiliations:** 1 Universidade Federal do Rio Grande do Norte – Instituto de Química – Natal (RN) – Brazil.; 2 Universidade Federal do Rio Grande do Norte – Departamento de Biomedicina – Natal (RN) – Brazil.; 3 Universidade Federal do Rio Grande do Norte – Programa de Pós-Graduação em Ciências da Saúde – Natal (RN) – Brazil.; 4 Universidade Federal do Rio Grande do Norte – Programa de Pós-graduação em Biologia Funcional e Estrutural – Natal (RN) – Brazil.; 5 Universidade Federal do Rio Grande do Norte – Departamento de Morfologia – Natal (RN) – Brazil.; 6 Universidade Federal do Rio Grande do Norte – Programa de Pós-Graduação em Ciências Odontológicas – Natal (RN) – Brazil.; 7 Universidade Federal do Rio Grande do Norte – Programa de Pós-graduação em Ciências Farmacêuticas – Natal (RN) – Brazil.; 8 Universidade Federal do Rio Grande do Norte – Departamento de Biofísica e Farmacologia – Natal (RN) – Brazil.

**Keywords:** Quinoxalines, Apoptosis, Inflammation, Analgesia

## Abstract

**Purpose:**

Bioactive molecules are relevant to fight cancer and associated conditions. Quinoxaline is a privileged N-heterocycle, notably as anticancer agents. Herein, we report the evaluation of the quinoxaline derivatives DEQX and OAQX as anticancer agents, as well as in function of their anti-inflammatory and analgesic activities.

**Methods:**

Quinoxalines were synthesized and tested as anticancer agents based on cell viability and Annexin V-FITC apoptosis. Anti-inflammatory activity was evaluated from mouse carrageenan peritonitis and levels of interleukin (IL)-1β and tumor necrosis factor (TNF)-alfa for enzyme-linked immunosorbent assay. Hot-plate and acetic acid-induced writing test were employed to investigate analgesia.

**Results:**

Both reduced the Ht-29 cell viability in a dependent-concentration manner (*p* < 0.001). Total apoptosis was detected for cells treated with 12.5 and 25 µg/mL of both the compounds for 24 and 48 h (all doses, p < 0.0001). DEQX (all doses, *p* < 0.01) and OAQX (all doses, p < 0.001) acted in leukocyte migration and decreased the IL-1β and TNF-β levels (*p* < 0.05). DEQX (all doses, *p* < 0.05) and OAQX (5mg/kg, *p* < 0.001) showed peripheral analgesic effect.

**Conclusions:**

*In-vitro* and *in-vivo* results suggest that these quinoxalines are promising for application in pharmacological area due to their anticancer, anti-inflammatory, and peripheric analgesia.

## Introduction

Cancer is one of the most common and deadly non-communicable diseases of the 21^st^ century, and intensive efforts have been devoted to researching new therapeutic products^1^. Nowadays, with the advances in medicinal chemistry, targeted strategies have emerged in the last decades as promising solutions to overcome the challenges of oncology treatments^2^^–^ 6. Apoptosis resistance is one of the most important hallmarks of cancer, and many investigations have indicated that mitochondrial dysfunction is involved in this process^7^^–^ 12. Phosphatidylserine (PS) is a phospholipid naturally present in the cellular membrane of healthy cells; when exposed to an external leaflet of the plasma membrane, it can act as apoptosis signaling to the immune system^13^. Therefore, the annexin V binding assay is directly related to cancer studies^14^^,^ 15.

Nitrogen heterocycles are key constituents in many biological reactions and synthetic bioactive molecules, and these features have driven the advances in drug development^16^. Quinoxaline is solidified as a privileged *N*-heterocycle in biological fields, especially in medicinal chemistry, due to its presence in many bioactive structures against different pathologies, notably as anticancer agents based on their *in-vitro* and *in-vivo* activities, as well as *in-silico* studies, and involving different mechanisms of action^17^^–^ 29.

**Figure 1:**
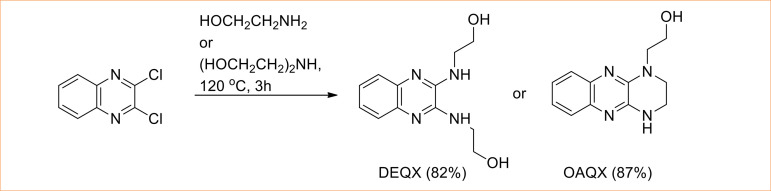
Synthesis of DEQX and OAQX from 2,3-dichloroquinoxaline.

Source: Elaborated by the authors.

Based on the role of simple quinoxaline derivatives as a promissing target for cancer treatment, this paper reports anticancer activity of two quinoxaline derivatives, DEQX and OAQX (Fig. 1), which were previously considered as anticancer agent, but they have never been tested for biological purposes of any nature^23^. The *in-vitro* antitumor activity of DEQX and OAQX was evaluated by cell viability using a colorectal cancer cell line (Ht-29), and annexin V and propidium iodide labeling were employed to evaluate cell death. In addition, as inflammatory responses are indicated as a quite relevant factor in different stages of tumor development^30^^,^ 31, the effect of both quinoxaline derivatives on mouse carrageenan peritonitis and levels of pro-inflammatory interleukin (IL)-1 β and tumor necrosis factor (TNF)-α were evaluated. Lastly, the central and peripheral analgesic activities of DEQX and OAQX were investigated.

## Methods

### Synthesis of quinoxaline derivatives

Compounds DEQX and OAQX were synthesized according previously reported, and spectroscopic data are coherent to their molecular structures^23^.

### In-vitro antitumor activity

The following reagents were purchased as indicated: Dulbecco’s modified Eagle’s medium (DMEM, Life Technologies, Grand Island, NY, United States of America), 10% (v/v) heat-inactivated fetal bovine serum (CULTILAB LTDA/Brazil), trypsin/EDTA (Gibco BRL, Life Technologies, Grand Island, NY, United States of America), and cisplatin (citoplax, 50 mg, Bergamo Taboão da Serra, SP, Brazil). A colorectal cancer cell line (Ht-29) was purchased from the Culture Collection of the Universidade Federal do Rio de Janeiro (RJCB Collection, Rio de Janeiro, RJ, Brazil). Ht-29 cells were maintained in Dulbecco’s modified Eagle’s medium supplemented with 10% (v/v) heat-inactivated fetal bovine serum. Molecules of DEQX and OAQX were diluted in DMSO (1%) and cisplatin diluted in medium (DMEM). All the solutions were filtered using a 0.22-mm minipore membrane, then were aliquoted and stored at -20 °C.

### Cell viability

Due to the different sensitivity of cancer cells (1 × 105) to the molecules DEQX and OAQX, the optimal exposure time for each cell line was determined in a pilot study in order to obtain a dose-dependent effect. The DMSO (1%) vehicle of DEQX and OAQX was also tested. Viability was determined at 24 h for Ht-29, and different concentrations (3.125–200 μg/mL in aqueous suspensions) of molecules were placed into the wells. Cell viability was determined by trypan blue exclusion assay. Brieﬂy, cell aliquots were mixed with same volume of 0.5% (w/v) trypan blue and incubated at room temperature for 5 minutes. The number of viable cells was calculated using a hemocytometer. Data was analyzed using GraphPad 5.0 (Prism Software, United States of America) to determine interval of confidence of 50 (IC50).

### Annexin V and propidium iodide staining

Cells were plated in 6-well plates (5 × 10^5^ cells/well) with 2 mL medium/well (triplicate). After 24 h, concentrations of both the compounds (3.125, 6.25, and 12.5 µg/mL) and cisplatin (50 and 100 µM) were added for 24 and 48 h. In parallel, control cells were maintained in culture medium without molecules and cisplatin. The cells were then assayed using the annexin V-FITC apoptosis detection kit I (Biosciences Pharmingen, San Diego, CA, United States of America). Annexin V-FITC and propidium iodide (PI) were added to the cellular suspension according to the manufacturer’s instructions. Each sample of cell line were then analyzed using a FACS Calibur cytometer (BD Bioscience, Franklin Lakes, NJ, United States of America) and FlowJo software (BD Biosciences). Annexin VFITC-positive/PI-negative cells were identified as cells in the early stages of apoptosis, while annexin V-FITC-positive/PI-positive cells were identified as cells in the late stages of apoptosis, or cells that were undergoing necrosis.

### In-vivo experiments

#### Animals

Male and female Swiss mice (25–35 g) and rats (200–250 g), respectively, were obtained from Bioterio of the Biophysical and Pharmacology Department. All animals were housed in an animal room under standard laboratory conditions of 22 ± 2 °C and 12-h light/12-h dark cycle and fed with pellet food and water *ad libitum*. They were acclimatized for seven days before the experiments started and fasted for 12 h prior to the experiments. Animal welfare and experimental procedures were in strict accordance with Ethics Committee on Animal Use, approved protocol no. 047/2013 and no. UFRN 047/2013.

### Drug administration

To determine their effects, compounds of DEQX and OAQX were suspended in DMSO before administration. Reagents: DMSO (VETEC Química, Rio de Janeiro, RJ, Brazil), indomethacin (INDOCID 25 mg Aspen Pharma), morphinesulphate (Dimorf 10 mg/mL^-1^/Cristália, São Paulo, SP, Brazil), and Diazepan (TEUTO, São Paulo, SP, Brazil).

### Anti-inflammatory activity

#### Mouse carrageenan peritonitis

The female rats were divided into eight groups (n = 6/group). Compounds of DEQX and OAQX were administered orally at doses of 0.5, 1 and 5 mg/kg. Positive control group was vehicle (10 mL/kg, p.o.). Indomethacin (10 mg/kg; p.o.) was included as a standard group. Carrageenan (Sigma-Aldrich) (0.25 mL, 1% in saline) was intraperitoneally injected 30 min later the treatment with compounds, vehicle and indomethacin, and after 4 h the animals were sacriﬁced by thiopental (100 mg/kg) for further investigation. The total leukocyte count was determined in a Neubauer chamber^32^.

### Interleukin-1β and tumor necrosis factor-α assay

Peritoneal fluid, stored at -70ºC after extraction, was homogenized and processed as previously described^33^. Levels of IL-1β (detection range: 62.5–4,000 pg/mL; lower limit of detection: 12.5 ng/mL recombinant mouse IL-1β), and TNF-α (detection range: 62.5–4,000 pg/mL; lower limit of detection: 50 ng/mL recombinant mouse TNF-α) were determined using commercial enzyme-linked immunosorbent assay (ELISA) kits (R&D Systems, Minneapolis, MN, United States of America), as previously described34. All samples were measured at 490 nm.

### Analgesic activity

#### Hot-plate test: central analgesic activity

The hot-plate test was carried out using a hot-plate apparatus (model Insight, São Paulo, SP, Brazil), maintained at 55 ± 0.5 °C. The male Swiss mice were divided into ﬁve groups of six animals each and were fasted overnight. Only mice that showed initial nociceptive responses (licking of the forepaws or jumping) between 3 and 19 were used for additional experiments. The chosen mice were pre-treated with compounds of DEQX and OAQX (0.5, 1 and 5 mg/kg; p.o.), and 30 min later the measurements were taken. Positive control group was treatment with vehicle (10 mL/kg, p.o.). A morphine group (10 mg/kg; i.p.) was included as a standard group. The cut-off time was set at 30 seconds to minimize skin damage. The reaction time, *i.e.*, the amount of time it takes the animal to lick their forepaws or jump off its hot plate, was measured at 0, 30, 60, 90 and 120 min after the administration^35^. After, the animals were sacriﬁced by thiopental (100 mg/kg).

#### Acetic acid-induced abdominal writhing test: peripheral analgesic activity

The method of Koster et al. was used for this test^36^. The female Swiss mice (seven for group) were divided into ﬁve groups of six mice each and fasted overnight. The animals were treated with indomethacin (standard group, 10 mg/kg, p.o.), vehicle (10 ml/kg, p.o.) and compounds of DEQX and OAQX (0.5, 1 and 5 mg/kg, p.o.). The mice were treated with acetic acid (0.6%, v/v in saline, 10 mL/kg, i.p.) 30 minutes after the already-mentioned treatment was carried out. The number of writhes was counted for 20 min. Afterwards, the animals were sacriﬁced by thiopental (100 mg/kg).

### Experimental outcomes

The animals were paired for sex, weight, age. During treatment, all groups were evaluated coat, motor activity (open Field) and death. No changes were recorded between groups.

### Statistical analysis

All experiments were performed at least in triplicate, and significant differences between groups were calculated using analysis of variance (ANOVA) and Bonferroni’s test, as indicated. P < 0.05 was considered statistically significant.

## Results

### Chemistry

Quinoxaline derivatives DEQX and OAQX were obtained by reaction of previously prepared 2,3-dichloroquinoxaline (DCQX) and appropriate aminoalcohols, ethanolamine and diethanolamine, respectively, under heating for 3 hours (Fig. 1). The target compounds were obtained in good yielding, 82–87%, and infrared (IR), as well as 1H and 13C nuclear magnetic resonance (NMR) spectroscopies data, is in agreement with those reported in literature^23^.

### Cytotoxicity

**Figure 2:**
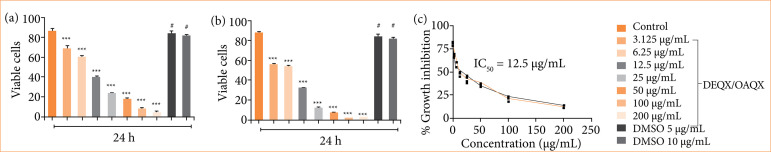
DEQX and OAQX reduced cell viability in a concentration-dependent manner after 24 h: **(a)** DEQX; **(b)** OAQX. Concentration range: 3.125–200 μg/mL. DMSO 1% was used as vehicle.

Ht-29 cells showed a decreased growth when they were submitted to lower concentrations of DEQX and OAQX. According to triplan blue assay results, the concentrations of DEQX and OAQX significantly affected on cell viability in a concentration-dependent manner (3.125–200 µg/mL). As presented in Figs. 2a-b, the lowest mortality rate was obtained for DEQX and OAQX at concentrations of 3.125 (*p* > 0.0001), whereas the highest mortality rate was obtained at 200 µg/mL (*p* < 0.0001). In addition, DEQX and OAQX were cytotoxic on Ht-29 cells with a half-maximal inhibiting concentration value (IC50) of 12.5 µg/mL, (*p* < 0.05) (Fig. 2c). However, the concentrations 6.25 and 25 µg/mL showed death percentage near to IC50. DMSO (5 µg/mL) did not alter the growth of Ht-29 growth.

***p < 0.0001; ^#^ *p* > 0.05. Source: elaborated by the authors.

### Evaluation apoptosis induction by DEQX and OAQX using flow cytometry

**Figure 3:**
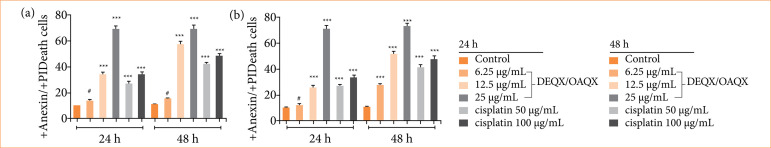
Detection of total apoptosis induced by **(a)** DEQX and **(b)** OAQX (12.5–25 μg/mL) and cisplatin (50 and 100 μM) for 24 and 48 h using flow cytometry.

**Figure 4:**
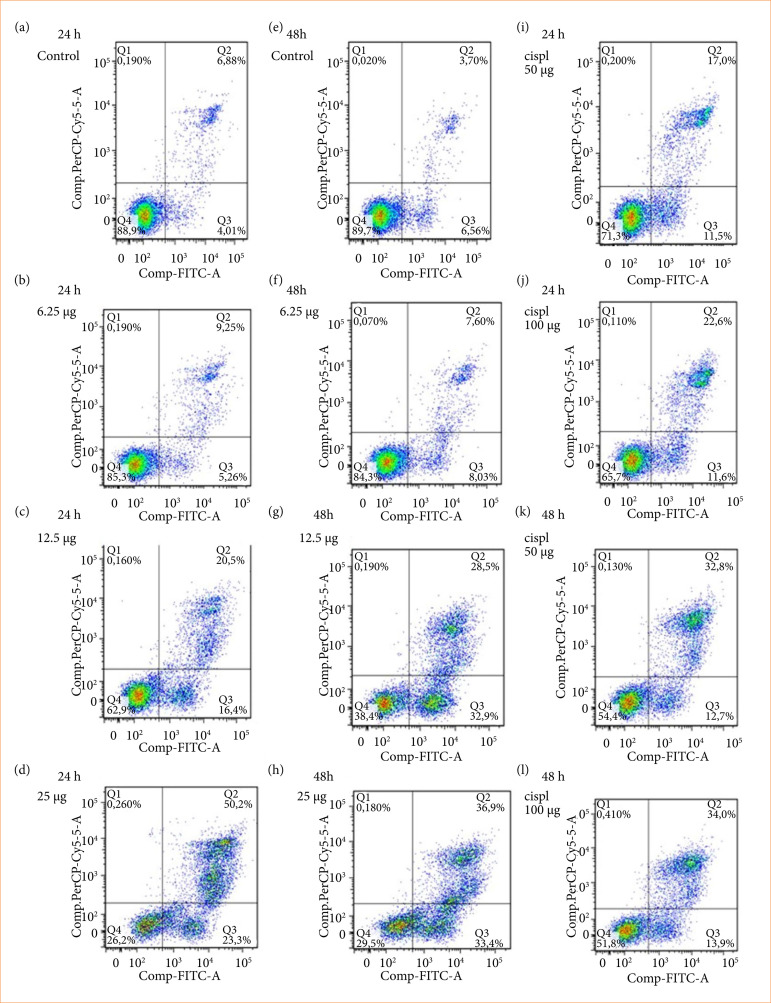
Effects of DEQX (12.5–25 μg/mL) and cisplatin (50 and 100 μM) on early and late apoptosis of Ht-29 cell lines as detected using flow cytometry.

Source: Elaborated by the authors.

**Figure 5:**
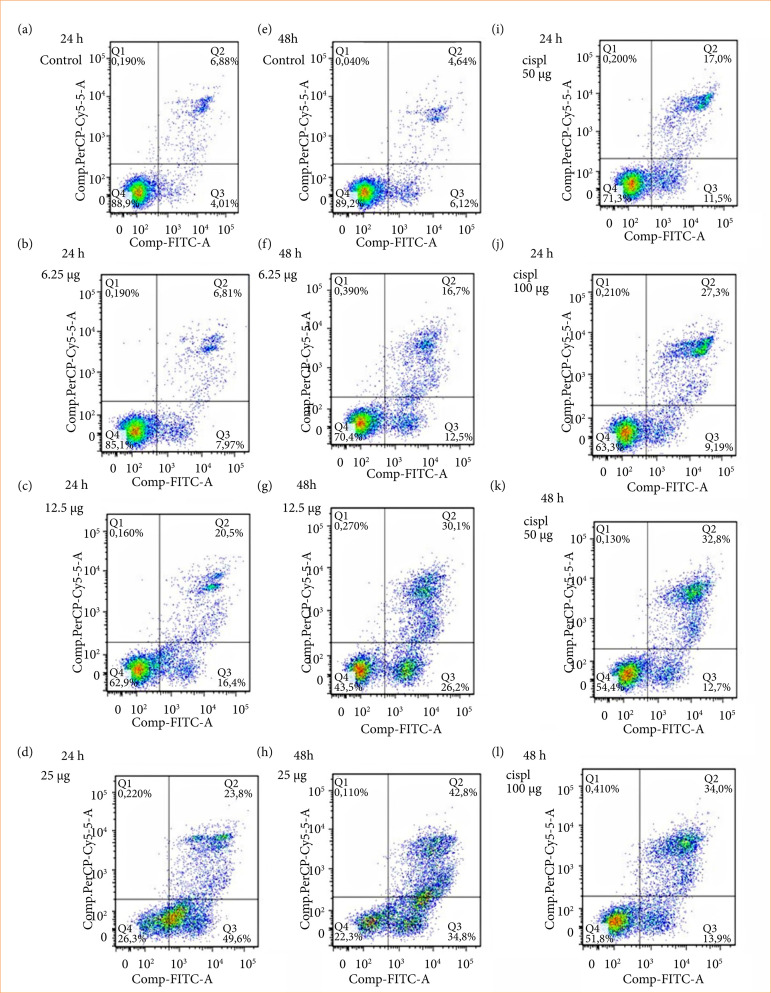
Effects of OAQX (12.5–25 μg/mL) and cisplatin (50 and 100 μM) on early and late apoptosis of Ht-29 cell lines as detected using flow cytometry.

Source: Elaborated by the authors.

To determine whether cell death induced by DEQX and OAQX was achieved through apoptosis, cells were treated with referred quinoxaline derivatives and stained with Annexin-V-FITC and PI. Using flow cytometry, early and late stages of apoptosis were detected based on the percentage of annexin VFITC-positive cells/PI-negative cells and the percentage of annexin V-FITC-positive/PI-positive cells that were present, respectively (Figs 3, 4 and 5), lower right quadrant data versus top left quadrant data, respectively.

For Ht-29 cells, Figures 4 (a-e) and 5 (a-e) show control group treatment with 6.25, 12.5, and 25 µg/mL of DEQX compound induced early and late apoptosis either to 24 or 48 h after treatment (Figs. 3a and 4b-g). When Ht-29 cells were treated with 6.25, 12.5, and 25 µg/mL by OAQX compound, it also induced early and late apoptosis both 24 and 48 h after treatment (Fig. 3b and 5b–5g). Ht-29 cells were treated with Ht-29 cell (50 and 100 µM), and both the early and late stage apoptosis were detected 24 and 48 h after treatment. However, a higher percentage of cells in the late stages of apoptosis were observed (Fig. 3, 4i-j and 5i-j).

### Anti-inflammatory effect in model carrageenan-induced peritonitis

**Figure 6:**
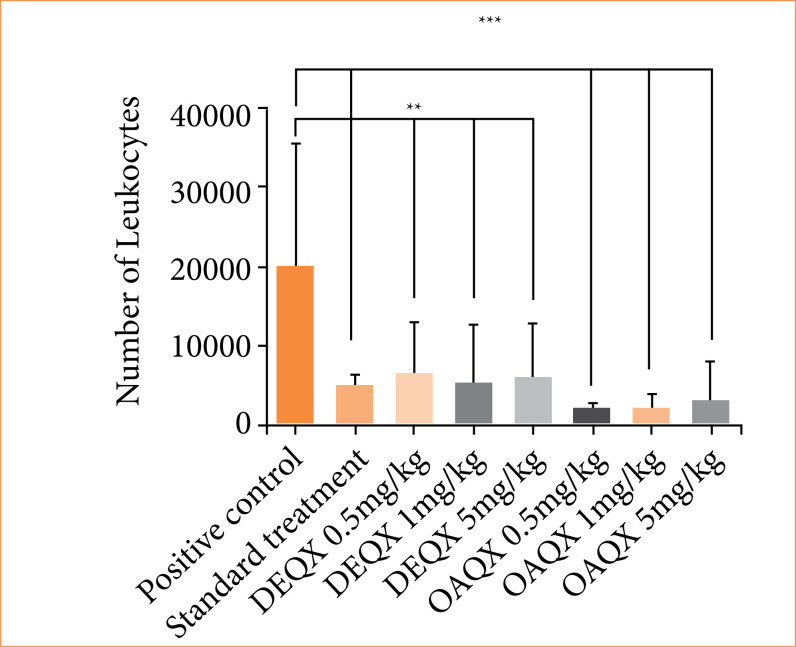
Cell migration treatment by DMSO/positive control, indomethacin/standard treatment, DEQX (0.5, 1 and 5 mg/kg), and OAQX (0.5, 1 and 5 mg/kg) (analysis of variance test: *p < 0.05). The bars represent the mean number of leucocytes + standard deviation groups compared with positive control (DMSO).

Source: Elaborated by the authors.

In order to evaluate a possible inhibitory effect of DEQX (*p* < 0.01) and OAQX (*p* < 0.001), the carrageenan-induced peritonitis test was used on all doses on cell recruitment into the peritoneal cavity (Fig. 6). The negative control group showed an increase in the numbers of leukocytes from peritoneal exudates, and indomethacin showed an inhibitory effect on cell recruitment into the peritoneal cavity (*p* < 0.001).

### Effects on inflammatory activity levels of interleukin-1β and tumor necrosis factor-α

**Figure 7:**
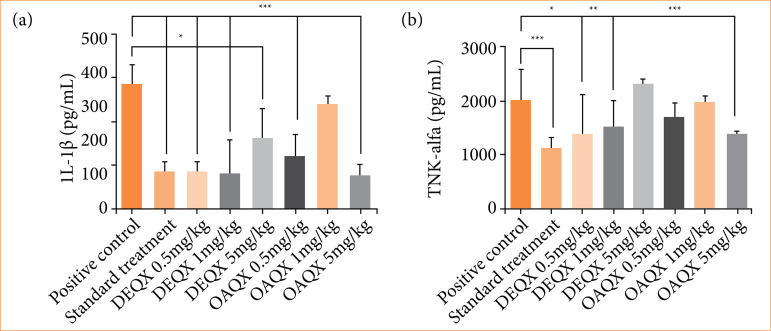
Levels of **(a)** interleukin (IL)-1β and **(b)** tumor necrosis factor (TNF)-α treatment by saline (no peritonitis); DMSO (with peritonitis); DEQX (0.5, 1 and 5 mg/kg; with peritonitis); OAQX (0.5, 1 and 5 mg/kg; with peritonitis). Anallysis of variance test: *p < 0.05; **p < 0.01; ***p < 0.001. Groups compared with DMSO group.

Source: Elaborated by the authors.

The group of DEQX decreased levels of IL-1β (0.5 and 1 mg/kg, *p* < 0.001; and 5 mg/kg, *p* < 0.05) compared to positive control (Fig. 7). Levels of anti-inflammatory cytokine IL-1β were decreased in the group treated with 0.5 and 5 mg/kg of OAQX compared to positive control (*p* < 0.001). Levels of TNF-α were decreased in animals treated with 0.5 and 1 mg/kg of DEQX, *p* < 0.05 and *p* < 0.001, respectively, compared to positive control. OAQX (5 mg/kg) showed reduced levels of TNF-α (*p* < 0.001) compared with positive control group. Levels of anti-inflammatory cytokine IL-1β and TNF-α were decreased in the indomethacin treatment compared to positive control (*p* < 0.001).

### Analgesic activity: hot-plate test and acetic acid writhing reflex

None of DEQX and OAQX (concentrations of 0.5, 1, and 5 mg/kg) presented central analgesic activity (p > 0.05) based on the hot-plate test, as presented in Table 1. In contrast, morphine showed central analgesic activity, for 30 minutes (*p* < 0.01), 60 minutes (*p* < 0.001), 90 minutes (*p* < 0.01), and 120 minutes (*p* < 0.01).

**Table 1:** Number pain latency time.

Groups	Pain latency at the indicated time after and before administration				
	0 s	30 min	60 min	90 min	120 min
Control (DMSO)	13.4 + 5.0	11.2 + 4.2	11.4 + 3.9	11.6+ 6.2	9.8 + 7.6
Morphine (10 mg/kg)	10 + 4.0	28 + 2.1**	25.3 + 3.0***	22.5 + 4.9**	22. 75 +2.6**
DEQX					
(0.5 mg/kg)	15 + 3.6	19 + 9.9	17 + 7.1	14.4 + 6.0	12.2 + 6.6
(1.0 mg/kg)	12 + 5.9	18 + 10.2	4.2 + 1.3	9.2 + 3.8	6.6 + 2.7
(5.0 mg/kg)	11.8 + 3.0	17.2 + 3.5	10.2 + 4.1	12.2 + 4	7.8 + 2.2
OAQX					
(0.5 mg/kg)	14.2 + 6.8	14.2 + 10.3	12.8 + 11.3	12.8 + 10.6	9 + 4.1
(1.0 mg/kg)	15.2 + 2.3	16.6 + 7.2	12.4 + 9.5	11.2+ 10.6	13.6 + 10.1
(5.0 mg/kg)	10.2 + 3.7	12.6 + 9.1	7.6 + 5.1	6.6 + 3.2	12 + 10.2

* Compared to the negative control group (DMSO); morphine (10 mg/kg).

Analysis of variance test:

** *p* < 0.01, *p* < 0.001.

Source: Elaborated by the authors.

**Figure 8:**
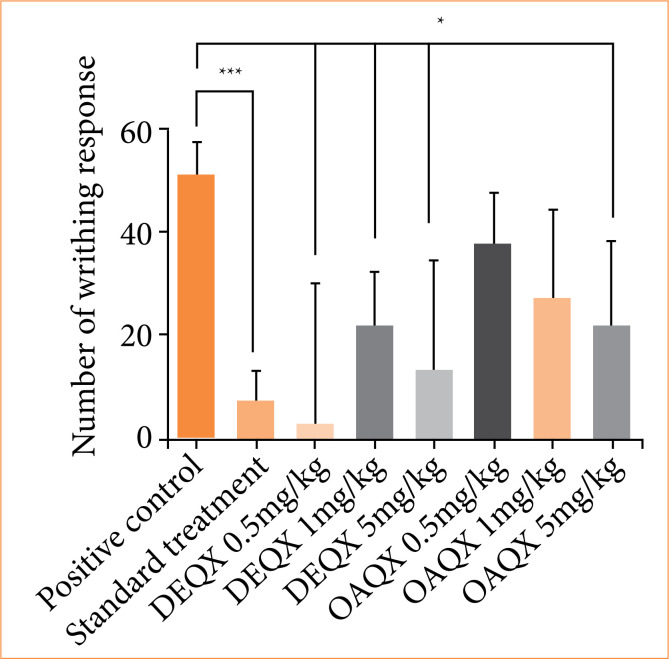
Number of writhing responses by positive control/DMSO, standard treatment/indomethacin, DEQX and OAQX. Analysis of variance test: *p < 0.05, ***p < 0.001. The bars represent the mean number of writhing + standard deviation, groups compared with positive control/DMSO.

Source: Elaborated by the authors.

The effect of quinoxaline derivatives on the writhing response in mice is shown in Fig. 8. Both quinoxaline derivatives, DEQX (all doses) and OAQX (5 mg/kg) decreased the writhing response (*p* < 0.05). In addition, indomethacin significantly decreased the writhing response (*p* < 0.001). Although a decreasing in the writhing response was observed for both quinoxaline derivatives in different doses, better results were verified for DEQX.

## Discussion

The compound DCQX is a versatile building block in organic synthesis due to its reactivity toward different nucleophilic species, and this feature has been employed for obtainment of several bioactive quinoxalines^22^. Reactions of DCQX with ethanolamine and diethanolamine lead to DEQX and OAQX in good yielding and high purity without the need of laborious purification procedures. While DEQX was obtained by double *N*-substitution by action of ethanolamine, the OAQX was formed by *N,O*-nucleophilic attack of diethanolamine, including a intramolecular reaction. Compounds were full characterized by IR, 1H NMR and 13C NMR techniques, as well as elemental analysis, and the data are coherent to proposed structures. In general, solubility of quinoxaline derivatives in water is very poor. However, both DEQX and OAQX were found to be fairly soluble in water and highly soluble in DMSO.

The PS that is externalized not only contributes to the recognition and subsequent removal of apoptotic bodies by phagocytes^37^, but also provides a binding site for the anionic lipid binding protein annexin V^38^, which is widely used to detect apoptotic cells. In addition to its use in laboratory studies *in vitro*, annexin V binding is being explored as a potential early marker of treatment efficacy in cancer patients^39^. Inour study, DEQX and OAQX showed anti-tumor activity by blocking cell viability and inducing apoptosis in colorectal cancer cells. Both compounds act in two stages of apoptosis, causing the externalization of PS in cell membrane. Both DEQX and OAQX had greater anti-tumor activity than standard anti-neoplastic cisplatin, thereby showing greater power of action. Previous studies showed that quinoxaline derivatives are potent hypoxic cytotoxin and pro-apoptotic drug in several murine and human cancer cell lines^40^^–^ 43. On the other hand, some quinoxaline derivatives reported in literature, such as compound WR23, have their anti-tumor activity hypothesized on binding to enzyme P3Kiα^44^^,^ 45. In this context, due the structural similarity to WR23 besides some additional polifunctionalyzed groups contained in DEQX and OAQX, it is suggested that DEQX and OAQX are able to establish a network of H-bond interactions by binding aminoacid sites in the enzyme^23^.

The literature brings interesting examples of quinoxaline derivatives having anti-inflammatory and analgesic activities^17^^,^ 28 ^,^ 29. One effect that has been observed for both DEQX and OAQX was anti-inflammatory activity, which reduces leucocitary migration and chemotaxis inhibition. This action led to significant reduction in the levels of pro-inflammatory IL-1 β and TNF-α, and peripheral analgesic activities were found for both quinoxaline derivatives, which corroborates this finding. A plausible explanation consists in the possibility of compounds DEQX and OAQX acting as non-peptide small molecule antagonists of IL-1 β and TNF-α receptors. However, further studies are needed to prove this hypothesis. Compounds DEQX and OAQX were tested as potential analgesics based on hot-plate test and acetic acid writhing reflex assays. A decreasing in the writhing response was observed for both quinoxaline derivatives in different doses, and better results were verified for DEQX.

## Conclusion

The synthetic quinoxaline small molecules DEQX and OAQX were able to reduce the Ht-29 cell viability in a dependent-concentration (*p* < 0.001). Moreover, the total apoptosis was detected for cells treated with 12.5 and 25 µg/mL of both the compounds for 24 and 48 h (all doses, *p* < 0.0001). Aiming anti-inflammatory activity, results showed that DEQX (all doses, *p* < 0.01) and OAQX (all doses, *p* < 0.001) acted in leukocyte migration and decreased the IL-1β and TNF-β levels (*p* < 0.05). Lastly, DEQX (all doses, *p* < 0.05) and OAQX (5 mg/kg, *p* < 0.001) showed peripheral analgesic effect.

In summary, *in-vitro* and *in-vivo* results suggest that quinoxaline derivatives DEQX and OAQX are promissing for application in pharmacological area due to their anticancer, anti-inflammatory and peripheric analgesia.

## Data Availability

All data sets were generated or analyzes in the current study

## References

[1] Neves AC de, Araújo Júnio, Oliveira ALC de, Lima KMG (2014). The use of EEM fluorescence data and OPLS/UPLS- DA algorithm to discriminate between normal and cancer cell lines: a feasibility study. Analyst.

[2] Sparreboom A, Jonge MJA De, Verweij JJ (2002). The use of oral cytotoxic and cytostatic drugs in cancer treatment. Eur J Cancer.

[3] Dilda PJ, Hogg PJ (2007). Arsenical-based cancer drugs. Cancer Treat Rev.

[4] Vuuren RJ, Visagie MH, Theron AE, Joubert AM (2015). Antimitotic drugs in the treatment of cancer. Cancer Chemother Pharmacol.

[5] Zheng Y, Chang X, Huang Y, He D (2023). The application of antidepressant drugs in cancer treatment. Biomed Pharmacother.

[6] Liu Y-P, Zheng C-C, Huang YN, He ML, Xu W-W, Li B. (2021). Molecular mechanisms of chemo- and radiotherapy resistance and the potential implications for cancer treatment. Med Commun.

[7] Ogbourne SM, Suhrbier A, Jones B, Cozzi S, Jones B, Boyle GM, Morris M, Mcalpine D, Johns J, Scott TM, Sutherland KP, Gardner JM, Le TTT, Lenarczyk A, Aylward JH, Parsons PG (2004). Antitumor Activity of 3-Ingenyl Angelate: Plasma Membrane and Mitochondrial Disruption and Necrotic Cell Death. Cancer Res.

[8] Rotem R, Heyfets A, Fingrut O, Blickstein D, Shaklai M, Flescher E (2005). Jasmonates: Novel Anticancer Agents Acting Directly and Selectively on Human Cancer Cell Mitochondria. Cancer Res.

[9] Wang J-Q, Zhang P-Y, Qian C, Hou X-J, Ji LN, Chao H (2023). Mitochondria are the primary target in the induction of apoptosis by chiral ruthenium (II) polypyridyl complexes in cancer cells. J Biol Inorg Chem.

[10] Park M, Kang KW (2019). Phosphatidylserine receptor-targeting therapies for the treatment of cancer. Arch Pharm Res.

[11] Chang W, Fa H, Xiao D, Wang J (2020). Targeting phosphatidylserine for Cancer therapy: prospects and challenges. Theranostics.

[12] Kaynak A, Davis HW, Kogan AB, Lee J huei, Narmoneva DA, Qi X (2022). Phosphatidylserine: The Unique Dual-Role Biomarker for Cancer Imaging and Therapy. Cancers.

[13] Birge RB, Boeltz S, Kumar S, Carlson J, Wanderley J, Calianese D, Barcinski M, Brekken RA, Huang X, Hutchins JT, Freimark B, Empig C, Mercer J, Schroit AJ, Schett G, Herrmann M (2016). Phosphatidylserine is a global immunosuppressive signal in efferocytosis, infectious disease, and cancer. Cell Death Differ.

[14] Lee SH, Meng XW, Flatten KS, Loegering DA, Kaufmann SH (2012). Phosphatidylserine exposure during apoptosis reflects bidirectional trafficking between plasma membrane and cytoplasm. Cell Death Differ.

[15] Liu T, Zhu W, Yang X, Chen L, Yang R, Hua Z, Li G (2009). Detection of Apoptosis Based on the Interaction between Annexin V and Phosphatidylserine. Anal Chem.

[16] Walsh CT (2015). Nature loves nitrogen heterocycles. Tetrahedron Letters.

[17] Cordeiro DS, Fernandes R, Pereira JA, Pessoa AM, Nat M, Prud C (2015). Quinoxaline, its derivatives and applications: A State of the Art review. Eur J Med Chem.

[18] Montana M, Mathias F, Terme T, Vanelle P (2019). Antitumoral activity of quinoxaline derivatives: A systematic review. Eur J Med Chem.

[19] Pinheiro AC, Nogueira TCM, Souza MVN (2016). Quinoxaline Nucleus: A Promising Scaffold in Anti-cancer Drug Discovery. Anti-Cancer Agents Med Chem.

[20] Suthar SK, Chundawat NS, Singh GP, Padrón JM, Jhala YK (2022). Quinoxaline: A comprehension of current pharmacological advancement in medicinal chemistry. Eur J Med Chem Rep.

[21] Neri JM, Latocheski E, Araújo JGL, Lima RP, Cavalcanti LN, Neves ACO, Domingos JB, Menezes FG (2021). Quinoxaline-functionalized silver nanoparticles as chromogenic probe for the multiple selective detection of cysteine, Mg2+ and Sn2+ in aqueous solution. Sens Actuators B Chem.

[22] Neri JM, Cavalcanti LN, Araújo RM (2020). 2,3-Dichloroquinoxaline as a versatile building block for heteroaromatic nucleophilic substitution: A review of the last decade. Arab J Chem.

[23] Araújo J, Menezes FG, Silva HFO, Vieira DS, Silva SRB, Bortoluzzi AJ, Sant´Anna C, Eugenio Neri, Gasparotto LHS (2019). Functionalization of gold nanoparticles with two aminoalcohol-based quinoxaline derivatives for targeting phosphoinositide 3-kinases (PI3Ka). New J Chem.

[24] Montana M, Montero V, Khoumeri O, Vanelle P (2020). Quinoxaline Derivatives as Antiviral Agents: A Systematic Review. Molecules.

[25] Khatoon H, Abdulmalek E (2021). Novel Synthetic Routes to Prepare Biologically Active Quinoxalines and Their Derivatives: A Synthetic Review for Hena Khatoon 1, the Last Two Decades. Molecules.

[26] Freitas GRS, Coelho SE, Monteiro NK, V Neri, Cavalcanti LN, Domingos JB, Vieira DS, Souza MAF, Menezes FG (2017). Theoretical and Experimental Investigation of Acidity of the Glutamate Receptor Antagonist 6,7-Dinitro-1,4-dihydroquinoxaline-2,3-dione and Its Possible Implication in GluA2 Binding. J Phys Chem A.

[27] Silva LC, Machado VG, Menezes FG (2021). Quinoxaline-based chromogenic and fluorogenic chemosensors for the detection of metal cations. Chem Pap.

[28] Meka G, Chintakunta R. (2023). Analgesic and anti-inflammatory activity of quinoxaline derivatives: Design synthesis and characterization. Results Chem..

[29] Abu-hashem AA, Gouda MA, Badria FA (2010). Synthesis of some new pyrimido[20,10:2,3]thiazolo[4,5-b]quinoxaline derivatives as anti-inflammatory and analgesic agents. Eur J Med Chem.

[30] Coussens LM, Werb Z (2002). Inflammation and cancer. Nature.

[31] Grivennikov SI, Greten FR, Karin M (2010). Immunity, Inflammation, and Cancer Sergei. Cell.

[32] Ribeiro RA, Flores CA, Cunha FQ, Ferreira SH (1991). IL-8 causes in vivo neutrophil migration by a cell-dependent mechanism. Immunology.

[33] Safieh-Garabedian B, Poole S, Allchorne A, Winter J, Woolf CJ (1995). Contribution of interleukin-1 to the inflammation-induced increase in nerve growth factor levels and inflammatory hyperalgesia. Brit J Pharmacol..

[34] Kendall C, Ionescu-Matiu I, Dreesman GR (1983). Utilization of the biotin/avidin system to amplify the sensitivity of the enzyme-linked immunosorbent assay (ELISA). J Immunol Methods.

[35] Kuraishi Y, Harada Y, Aratani S, Satoh M, Takagi H (1983). Separate Involvement of the Spinal Noradrenergic and Serotonergic Systems in Morphine Analgesia: the Differences in Mechanical and Thermal Algesic Tests. Brain Res.

[36] Koster R, Anderson M, Debeer E. (1959). J. Acetic acid for analgesic screening. Fed Proc.

[37] Zhou Z (2007). New phosphatidylserine receptors: clearance of apoptotic cells and more. Dev Cell.

[38] Zwaal RFA, Comfurius P, Bevers EM (2005). Surface exposure of phosphatidylserine in pathological cells. Cell Mol Life Sci.

[39] Neves AA, Brindle KM (2006). Assessing responses to cancer therapy using molecular imaging. Biochim Biophys Acta.

[40] Gali-Muhtasib HU, Haddadin MJ, Rahhal DN, Younes IH (2001). Quinoxaline 1,4-dioxides as anticancer and hypoxia- selective drugs. Oncol Rep.

[41] Diab M, Haddadin MJ, Yared P, Assaad C, Gali-muhtasib HU (2002). Quinoxaline 1,4-Dioxides: Hypoxia-Selective Therapeutic Agents. Mol Carcinog.

[42] Itani W, Geara F, Haykal J, Haddadin M, Gali-muhtasib H (2007). Radiosensitization by 2-benzoyl-3-phenyl-6,7-dichloroquinoxaline 1,4-dioxide under oxia and hypoxia in human colon cancer cells. Radiat Oncol.

[43] khatib M, Geara F, Haddadin MJ, Gali-muhtasib H (2010). Cell death by the quinoxaline dioxide DCQ in human colon cancer cells is enhanced under hypoxia and is independent of p53 and p21. Radiat Oncol.

[44] Wu P, Su Y, Liu X, Zhang L, Ye Y, Xu J, Weng S, Li Y, Liu T, Huang S, Yang B, He Q, Hu Y (2011). Synthesis and biological evaluation of novel 2-arylamino-3-(arylsulfonyl) quinoxalines as PI3K a inhibitors. Eur J Med Chem.

[45] Wu P, Su Y, Liu X, Yang B, He Q, Hu Y (2012). Discovery of novel 2-piperidinol-3-(arylsulfonyl)quinoxalines as phosphoinositide 3-kinase a (PI3Ka) inhibitors. Bioorg Med Chem.

